# Comparison of sampling methods for next generation sequencing for patients with lung cancer

**DOI:** 10.1002/cam4.4632

**Published:** 2022-03-10

**Authors:** Kei Kunimasa, Shingo Matsumoto, Kazumi Nishino, Keiichiro Honma, Noboru Maeda, Hanako Kuhara, Motohiro Tamiya, Takako Inoue, Takahisa Kawamura, Toru Kimura, Tomohiro Maniwa, Jiro Okami, Koichi Goto, Toru Kumagai

**Affiliations:** ^1^ Department of Thoracic Oncology Osaka International Cancer Institute Osaka Japan; ^2^ Department of Thoracic Oncology National Cancer Center Hospital East Kashiwa Japan; ^3^ Department of Diagnostic Pathology & Cytology Osaka International Cancer Institute Osaka Japan; ^4^ Department of Diagnostic and Interventional Radiology Osaka International Cancer Institute Osaka Japan; ^5^ Department of General Thoracic Surgery Osaka International Cancer Institute Osaka Japan

**Keywords:** lung cancer, next generation sequencing, NGS success rate, re‐biopsy, sampling method

## Abstract

**Introduction:**

Success of next generation sequencing (NGS) analysis is becoming indispensable in the treatment of advanced lung cancer. However, the advantages and disadvantages of each sampling method in the NGS analysis have not yet been clarified.

**Methods:**

We compared the success rates of NGS analysis, and DNA and RNA yields for transbronchial biopsy (TBB), endobronchial ultrasound‐guided transbronchial needle aspiration (EBUS‐TBNA), computed tomography (CT)‐guided biopsy, fluid sample, and surgical biopsy for NGS analysis in patients through the lung cancer genomic screening project for individualized medicine (LC‐SCRUM)‐Asia, a nationwide NGS screening project. In case, sufficient samples could not be collected by TBB and EBUS‐TBNA, re‐biopsy (genome re‐biopsy) was performed.

**Results:**

A total of 223 patients were enrolled and success rates of NGS analysis were not different between samples obtained through TBB, EBUS‐TBNA, and CT‐guided biopsy; however, success rates for fluid samples and surgical biopsy samples were significantly higher than those of other methods. The risk of genome re‐biopsy was higher with TBB for centrally located lesions. CT‐guided biopsy yielded more samples but had a lower success rate for analysis of RNA‐based NGS than TBB.

**Conclusions:**

TBB is the mainstay of sampling methods, but for centrally located lesions, EBUS‐TBNA may be a better strategy. For CT‐guided biopsy, the success rate of RNA‐based NGS analysis is low. Fluid samples are expected to yield successful results as surgical biopsy samples, but the latter are better for sample preservation. Determining the optimal method for genome biopsy for each case is important.

## Graphical abstract

We compared sampling methods for the successful pre‐treatment NGS analysis in LC‐SCRUM‐Asia in patients with advanced lung cancer. It is important to discern which sampling method is the best from the image and patient condition to avoid the re‐genome biopsy. The special attitude as a genome biopsy for the success of the NGS analysis is required instead of the biopsy for the diagnosis until now.

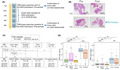

## 1 INTRODUCTION

Technological innovation in next generation sequencing (NGS) has led to dramatic advances in cancer genome research, and the accumulation of information on cancer genomes is increasing.^1^, ^2^, ^3^, ^4^ The identification of targetable driver mutations and the development and clinical application of molecular‐targeted therapies for non‐small cell lung cancer (NSCLC) is progressing rapidly, and the number of targetable driver mutations are steadily increasing when compared with that for other solid tumors.^5^, ^6^, ^7^ Starting with *EGFR* mutations,^8^, ^9^ clinical trials of molecular‐targeted drugs against *ALK* fusion genes,^10^, ^11^ *ROS1* fusion genes,^12^ *BRAF* mutations,^13^ *MET* exon 14 skipping mutations,^14^, ^15^ and *RET* fusion genes^16^, ^17^ have been conducted over the last 10 years. The identification of new targetable driver mutations and the development of corresponding drugs is expected to continue.^5^ Obtaining accurate and rapid information on the cancer genome through NGS analysis before the introduction of systemic treatment has become essential in treating NSCLC.

The Lung Cancer Genomic Screening Project for individualized Medicine (LC‐SCRUM) was launched in Japan in 2013 as a nationwide genomic screening project for lung cancer.^17^, ^18^, ^19^ More than 200 institutions in Japan have participated, and in the 7 years since the project began, more than 10,000 patients with lung cancer have been registered. Since 2019, the screening platform has been expanded to East Asia, and the scale of screening has been expanded as LC‐SCRUM‐Asia. NGS‐based genomic screening has been implemented since 2015, and the project also serves as the basis for the NGS database in lung cancer in Japan.^20^ NGS analysis of tumor tissue in patients with advanced lung cancer relies on obtaining bronchoscopic microspecimens for diagnosis because invasive procedures, such as surgery, are difficult to perform before treatment.^19^, ^21^, ^22^ Therefore, successful NGS analysis may be the first hurdle in the introduction of successful treatment. We realized improved success rate of NGS analysis in LC‐SCRUM‐Asia by improving bronchoscopy and sampling methods.^18^

In this study, we compared the sampling methods for NGS analysis in untreated advanced lung cancer and examined the improvement strategies of the sampling method with the aim of further improving the success rate of NGS analysis with no failure.

## 2 MATERIALS AND METHODS

### 2.1 Patients and clinical characteristics

LC‐SCRUM‐Asia, which was previously named LC‐SCRUM‐Japan, is a prospective, nationwide, clinical, and genomic screening program for lung cancer (UMIN ID: UMIN000010234). All patients provided written informed consent for the enrolment in the LC‐SCRUM‐Asia program. We retrospectively reviewed the records of all patients who were enrolled in LC‐SCRUM‐Japan at our institution. Since January 2019, our institute has improved and standardized sampling methods for NGS,^18^ and this study included all patients enrolled in LC‐SCRUM‐Japan from our hospital between January 2019 and December 2020.

Based on location, the primary lung tumor was categorized as central or peripheral using chest computed tomography (CT) imaging before treatment initiation. The criteria for categorization as central and peripheral were as follows^23^ central location was defined as within 2 cm of the proximal bronchial tree based on the Radiation Therapy Oncology Group (RTOG) criteria^24^ or within 2 cm of the heart, trachea, pericardium, or vertebral bodies, but 1 cm away from the spinal canal based on a modification of the MD Anderson Cancer Center definition.^25^ In addition, tumor localization and maximum diameter of the primary lesion in the lung were evaluated in the right and left upper or lower lobes, respectively.

### 2.2 Sampling methods for NGS analysis in LC‐SCRUM Asia

**FIGURE 1:**
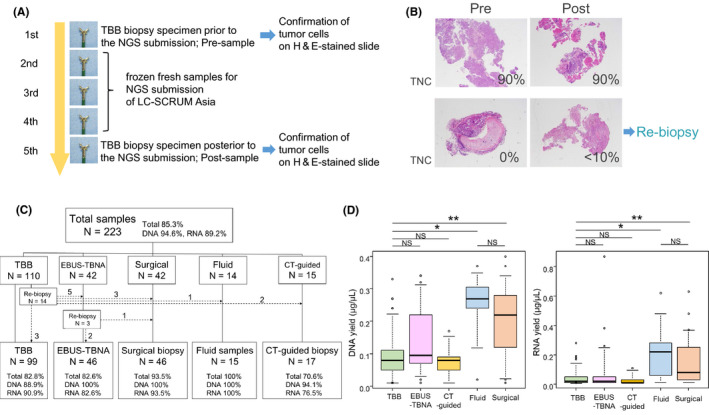
(A) Transbronchial biopsy (TBB) procedure. Five serial biopsies are performed, with the first and last samples subjected to histopathologic analysis and HE staining to identify tumor cells. The second to fourth biopsies were submitted to next generation sequencing (NGS) analysis as fresh frozen specimens. The frequency of biopsy varies from three to six times. (B) A representative confirmation slide showing images of TBB samples. Re‐biopsy was performed when the tumor nuclei content (TNC) was <10% for both pre‐ and post‐confirmation slides. (C) Classification of sampling methods for submitted specimens. A total of 223 samples were biopsied by five methods: transbronchial biopsy (TBB), endobronchial ultrasound with real‐time guided transbronchial needle aspiration (EBUS‐TBNA), surgical biopsy, fluid sample, and computed tomography (CT)‐guided biopsy. The success rate of NGS analysis is shown as a percentage, where “Total” is the percentage of success for both DNA‐based and RNA‐based NGS, “DNA” indicates the success rate of DNA‐based NGS, and “RNA” indicates the success rate of RNA‐based NGS. The dashed line represents the number of samples for re‐genome biopsy. (D) Comparison of DNA and RNA yields (μg/μl) among sampling methods. *p <0.05; **p <0.05. LC‐SCRUM‐Asia, lung cancer genomic screening project for individualized medicinel; TNC, tumor nuclei content

In LC‐SCRUM Asia, fresh frozen specimens obtained by each method were submitted. A total of 100 ml of body fluid specimens are also permitted. NGS analysis proceeds after the presence of tumor cells is confirmed. Sampling methods for NGS analysis, called “genome biopsy,” are being performed at our institution since January 2019 as follows: first, surgical biopsy specimens are preferentially submitted in collaboration with pathologists and surgeons; second, during sampling using transbronchial biopsy (TBB), standard biopsy forceps (FB‐231D.A; Olympus) with a 5.0‐mm cup opening were used^26^; third, during sampling using endobronchial ultrasound‐guided transbronchial needle aspiration (EBUS‐TBNA), whenever possible, biopsies were performed using a 19‐gauge needle at least twice; fourth, for CT‐guided biopsy, two samples were submitted; fifth, before and after sampling with transbronchial biopsy for NGS analysis, specimens were also collected for pathological analysis to confirm that the freshly obtained samples contained tumor cells (Figure 1A); finally, all samples were submitted following specimen evaluation by pathologists. In TBB, EBUS‐TBNA and CT‐guided biopsy sampling methods, we used rapid on‐site evaluation (ROSE) of tumor cells.^27^

For specimens collected through bronchoscopy, if the presence of viable tumor cells cannot be confirmed in both samples collected before and after the frozen samples to be submitted, or if viable tumor cell content is ≤10% (Figure 1B), NGS analysis is not expected to succeed, and the specimen will not be submitted (tissue confirmation). In these cases, a second biopsy, which is called “genome re‐biopsy,” was performed by changing the sampling method as necessary.

### 2.3 DNA and RNA extraction, NGS reports from LC‐SCRUM Asia and definition of analysis success or failure

DNA and RNA samples were extracted from fresh frozen specimens or from body fluids and analyzed using the Oncomine Comprehensive Assay v3 (OCA v3; Thermo Fisher Scientific), a targeted NGS assay, at the laboratories of SRL Incorporated. DNA/RNA were extracted and purified with a nucleic acid extraction kit (AllPrep DNA/RNA Mini Kit; Qiagen) according to the manufacturer's protocol. DNA/RNA concentrations were quantified by the Qubit fluorometric assay (Thermo Fisher Scientific).^19^ The minimum concentrations were defined as 1.67 ng/μl of DNA and 2.5 ng/μl of RNA. When the concentration was under the aforementioned levels, NGS analyses were not performed. The yields of DNA/RNA were reported to each institution.^19^ A target region containing 161 genes was amplified using multiplex PCR for DNA and RNA, and somatic mutations in the region were detected. Hotspot mutations (single nucleotide variants, deletions, and insertions) and copy number variations were detected in DNA‐based sequences, and fusion gene alterations were detected in RNA‐based sequences. The secretariat of LC‐SCRUM‐Asia reported the concentration of extracted DNA and RNA and the results of NGS analysis. Of the multiple somatic mutations analyzed using the OCA v3. panel, 20 mutations, which have been reported to be associated with the pathogenesis of lung cancer and have the corresponding therapeutic agents, including unapproved drugs, were reported to each attending physician. These mutations included *RET*, *ALK*, and *ROS1* fusion genes; *FGFR* 1–4 mutations, amplifications, and fusions; *MET* and *ERBB2* mutations and amplifications; *AKT1*, *BRAF*, *HRAS*, *KRAS*, *NRAS*, *EGFR*, and *PIK3CA* mutations; and *NTRK*1‐3 fusion genes and *NRG1* fusion gene. When the amounts of extracted DNA and RNA did not meet the criteria for NGS analysis, the amount of nucleic acid was reported insufficient. In this study, we defined analysis failure when NGS analysis was not accomplished for reasons including insufficient DNA or RNA sample volumes. DNA‐ and RNA‐based NGS analysis was determined to have succeeded and failed separately, and only the cases in which both were successful were evaluated as successful cases in this study.

### 2.4 Comparison of success rates of NGS analysis before and after introduction of the improved strategies

We compared the success rate of NGS analysis, the method of collecting submitted samples, and the yields of DNA and RNA before and after the introduction of the improvement strategies based on the electronic medical records and the secretariat reports.

### 2.5 Assessment of tumor nuclei, necrosis tissue contents, and sample size in confirmation slides

Two pathologists independently evaluated tumor cell and necrosis tissue contents of confirmation slides of pre‐ and post‐submitted samples and assessed tumor nuclei content (TNC) (%) and proportion of necrotic tissue area (%).^28^, ^29^ The mean values of tumor nuclei content (%) and necrotic tissue area (%) at the pre‐ and post‐slides were used for each case. Sample sizes of specimens obtained by TBB or CT‐guided biopsy in confirmation slides were measured using the free software ImageJ (Free soft, Image Processing and Analysis, http://rsb.info.nih.gov/ij).^26^ Regarding TBB samples, the average of the areas of samples in pre‐ and post‐slides was used as the area of the samples. For CT‐guided biopsy, the average of the areas of the two samples collected was used as the area of the samples.

### 2.6 Statistical analysis

Continuous variables were analyzed using Student's *t*‐test, and dichotomous variables were analyzed using χ^2^ or Fisher's exact test, as appropriate. All *p*‐values were two‐sided, with *p* < 0.05 considered statistically significant. To compare success rates between sampling methods, success rate analysis was performed with Fisher's exact test. Pairwise comparisons were determined using Fisher's exact test with Holm's adjusted *p*‐values. For comparing yields of DNA or RNA obtained from each sampling method, the Kruskal–Wallis and Steel–Dwass tests were used. Logistic regression analysis was used for multivariate analysis of factors related with genome re‐biopsy and NGS success rate in TBB samples; *p* < 0.05 was considered statistically significant. Statistical analyses were performed using EZR software ver 1.29 (Saitama Medical Center, Jichi Medical University, Saitama, Japan).^30^

## 3 RESULTS

### 3.1 Patient characteristics

A total of 223 patients from our institution were enrolled in LC‐SCRUM‐Asia. The clinical characteristics of the patients are summarized in Table 1. The median age was 67 years (range, 25–90 years). One hundred and twenty‐five patients (56.0%) were male, the majority (70.9%) had adenocarcinoma, including combined adenocarcinoma with squamous cell carcinoma or small cell carcinoma, and nearly half (47.5%) of the patients had the clinical stage of IVB, while the rest were almost equivalent in stage IIIA or B (24.7%) and IVA (27.8%). The targetable driver mutations reported by LC‐SCRUM‐Asia were detected in 120 (53.8%) out of 223 patients. The details of the reported mutations are shown in Table 1. During the course of this study, there were no insurance applicable drugs in Japan for *RET* fusion genes, *EGFR* Ex.20 insertion mutations, and *KRAS* G12C mutation. However, all four patients with *RET* fusion genes, one patient with *EGFR* Ex.20 insertion mutation, and two patients with *KRAS* G12C mutation were successfully enrolled in the clinical trials for corresponding driver mutations.

**TABLE 1:** Patients' characteristics

	Enrolled patients (*n* = 223)	(%) [total]
Age‐median (range)		
Median	67 (25–90)	
Sex, *n* (%)		
Male	125	(56.0)
Female	98	(44.0)
Histology, *n* (%)		
Ad including		
Ad+SCLC	158	(70.9)
Ad+Sq		
Sq	36	(16.1)
NSCLC	29	(13.0)
Stage‐n. (%)		
IIIA,B	55	(24.7)
IVA	62	(27.8)
IVB	106	(47.5)
Targetable driver mutations		
EGFR	[53]	[23.8]
Ex.19 deletion	27	(12.1)
L858R	21	(9.4)
T790M	2	(0.9)
L861Q	1	(0.5)
Ex.20 insertion	2	(0.9)
ALK fusion		
EML4‐ALK	9	(4.0)
ROS1 fusion	[5]	[2.2]
CD74‐ROS1	1	(0.4)
SDC4‐ROS1	2	(0.9)
SLC34A2‐ROS1	2	(0.9)
BRAF V600E	1	(0.4)
MET	[8]	[3.5]
Ex.14 skipping	5	(2.2)
Amplification	3	(1.3)
RET fusion		
KIF5B‐RET	4	(1.8)
HER2 Ex.20 ins	2	(0.9)
KRAS	[24]	[10.8]
G12A	2	(0.9)
G12C	10	(4.5)
G12D	9	(4.1)
G12V	2	(0.9)
Q61H	1	(0.4)
NRAS	[2]	[0.8]
Q61K	1	(0.4)
Q61L	1	(0.4)
PIK3CA	[4]	[1.8]
E545K	2	(0.9)
H1047R	2	(0.9)
NRG1‐CD74 fusion	1	(0.4)
FGFR1 amplification	4	(1.8)
FGFR3‐TACC3 fusion	3	(1.3)

Abbreviations: Ad, adenocarcinoma; Sq, squamous cell carcinoma.

### 3.2 Comparison of the sampling methods for NGS analysis in LC‐SCRUM Asia

The details of the sampling method for 223 patients are shown in Figure 1C. Among the specimens of the 110 patients in the TBB group, 14 required genome re‐biopsy. Of the specimens of the 42 patients in the EBUS‐TBNA group, three required genome re‐biopsy. Of the final specimens submitted, including re‐biopsy specimens, 99 (44.3%), 46 (20.6%), 46 (20.6%), 17 (7.6%), and 15 (6.9%) were obtained through TBB, EBUS‐TBNA, surgical biopsy, CT‐guided biopsy, and fluid specimens, respectively. The median yields of DNA and RNA obtained from each sampling method were 80 (10–33) ng/μl and 20 (10–28) ng/μl from TBB, 10 (10–340) ng/μl and 20 (10–870) ng/μl from EBUS‐TBNA, 220 (10–400) ng/μl and 80 (10–630) ng/μl from surgical biopsy, 80 (10–170) ng/μl and 10 (10–110) ng/μl from CT‐guided biopsy, and 270 (20–370) ng/μl and 220 (10–620) ng/μl from fluid samples, respectively. The median total amount of DNA and RNA extracted was as follows; DNA/RNA; 4.0/ 1.0 μg from TBB, 5.0/1.0 μg from EBUS‐TBNA, 11.0/4.0 μg from surgical biopsy, 4.0/0.5 μg from CT‐guided biopsy, and 13.5/11.0 μg from fluid samples. Success rates for DNA‐based, RNA‐based, or integrated NGS analysis are shown in Figure 1C. There was a significant difference in the success rate of DNA‐based NGS analysis (*p* = 0.013) between the sampling methods, but no significant difference was observed in RNA‐based and integrated NGS analysis (Table S1). However, the success rate of RNA‐based NGS analysis tended to be lower in CT‐guided biopsy samples than in TBB, fluid, and surgical biopsy samples. In integrated NGS analysis, success rates of TBB and CT‐guided biopsy samples tend to be lower than that of fluid and surgical biopsy samples (Table S1).

For groups that succeeded in DNA‐ or RNA‐based NGS analysis, significantly higher DNA and RNA yields were obtained from the samples (Figure S1). A comparison of each sampling method revealed no difference in DNA and RNA yields between TBB, EBUS‐TBNA, and CT‐guided biopsy sample groups; however, DNA and RNA yields tended to be significantly lower in these groups than in fluid and surgical biopsy sample groups (Figure 1D). Yields of DNA and RNA and success rate of NGS analysis were not significantly different between the fluid and surgical biopsy sample groups (Figure 1D) (Table S2).

### 3.3 Clinical factors associated with the risk of re‐genome biopsy and NGS analysis success in TBB samples

The clinical characteristics of 110 patients who had genome biopsy with TBB and 99 patients who finally underwent NGS analysis with TBB samples after genome re‐biopsy are shown in Table 2. We examined the clinical risk factors associated with re‐genome biopsy in 14 patients, who required a second genome biopsy, out of 110 patients. Results from univariate analysis revealed that only tumor location (peripheral or central) (*p* = 0.015) was significantly correlated with the risk of re‐genome biopsy (Table S3; Figure S2). Univariate analysis of the relationship between success rate of NGS analysis in TBB samples and clinical factors revealed that maximum diameter of tumor (ORR, 0.96; 95% CI, 0.924–0.998; *p* = 0.040) and tissue confirmation (ORR, 11.9; 95% CI, 3.480–40.60; *p* <0.001) are significantly correlated with NGS success rate. After multivariate analysis, only tissue confirmation remained a significant factor (Table 3).

**TABLE 2:** Clinical characteristics of patients who underwent genome biopsy with TBB

	Final TBB patients (*n* = 99)
Age‐median, (range)	
Median	67 (32–90)
Sex, *n* (%)	
Male	58 (58.6)
Female	41 (41.4)
Histology, *n* (%)	
Ad including	
Ad + SCLC	75 (75.8)
Ad + Sq	
Sq	16 (16.1)
NSCLC	8 (8.1)
Stage, *n* (%)	
IIIA,B	20 (20.2)
IVA	32 (32.3)
IVB	47 (47.5)
Tumor location 1	
Central	34 (34.3)
Peripheral	65 (65.7)
Tumor location 2	
Right	
Upper and Middle	29 (29.3)
Lower	24 (24.2)
Left	
Upper	24 (24.2)
Lower	22 (22.3)
Re‐biopsy for genome analysis	0 (0)
Maximum diameter of tumor (mm)	
Median (range)	40.3 (14.7–102.3)
Tumor nuclei content (%)	
Median (range)	50 (10–90)
Necrosis tissue content (%)	
Median (range)	10 (0–90)
Unconfirmed specimens	16 (16.2)

**TABLE 3:** Analysis of clinical and pathological factors for the success of NGS analysis in TBB samples

Variables	*n* = 99 (%)	Univariate		Multivariate	
		ORR (95% CI)	*p*	ORR (95% CI)	*p*
Age, years					
≤64	(35.4)	1	Reference		
>64	(64.6)	1.020 (0.343–3.050)	0.968		
Sex					
Male	(58.6)	1	Reference		
Female	(41.4)	0.717 (0.242–2.130)	0.549		
Histology					
Adenocarcinoma	(75.8)	1	Reference	1	Reference
Others	(24.2)	0.158 (0.019–1.25)	0.081	0.218 (0.020–2.36)	0.210
Tumor location 1					
Peripheral	(65.7)	1	Reference		
Central	(34.3)	0.747 (0.239–2.330)	0.616		
Tumor location 2					
Right upper/lower	(53.5)	1	Reference		
Left upper/lower	(46.5)	0.772 (0.477–1.250)	0.291		
Maximum diameter of tumor (mm) (range)					
40.3 (14.7–102.3)	(100)	0.96 (0.924–0.998)	0.040	0.98 (0.934–0.1.03)	0.358
Tumor nuclei content (%) (range)					
50 (10–90)	(100)	2.020 (0.702–5.790)	0.192	1.380 (0.401–4.73)	0.611
Necrosis tissue content. (%) (range)					
10 (0–90)	(100)	0.204 (0.025–1.640)	0.135	0.390 (0.038–4.02)	0.429
Tissue confirmation					
Confirmed	(83.8)	1	Reference	1	Reference
Unconfirmed	(16.2)	11.9 (3.480–40.60)	<0.001	11.1 (2.960–41.80)	<0.001

### 3.4 Comparison of the yields of DNA and RNA, and the success rates of NGS analysis between TBB and CT‐guided biopsy samples

We evaluated and compared clinical and imaging features and pathological features, such as TNC, necrosis tissue content, and tissue area, of the confirmation slide, in patients who submitted TBB (*n* = 99) and CT‐guided biopsy (*n* = 17) samples (Table 4). The lesions that were subjected to CT‐guided biopsy were located in the periphery and tended to have a larger maximum diameter than lesions that were subjected to TBB (median 49.8 vs. 40.3 mm, *p* = 0.076). Histopathological analysis of the confirmation slide revealed that in CT‐guided biopsy samples, the tumor area was significantly larger (median 5.6 vs. 1.7 mm^2^, *p* <0.001) and TNC was higher (median 70% vs. 50%, *p* <0.05) than in TBB samples. The yields of DNA and RNA were not different, but the concentrations of DNA and RNA per unit area were significantly lower in CT‐guided biopsy samples than in TBB samples (*p* < 0.001, respectively). These analyses indicate that CT‐guided biopsy tends to yield less DNA and RNA than TBB, even though CT‐guided biopsy yields larger samples containing more tumor cells than TBB.

**TABLE 4:** Comparison of clinical characteristics of patients who underwent genome biopsy TBB and CT‐guided biopsy

	Final TBB patients	Final CT‐guided patients	*p*
	(*n* = 99)	(*n* = 17)	
Age‐median, (range)			
Median	67 (32–90)	69 (36–82)	0.401
Sex, *n* (%)			
Male	58 (58.6)	8 (47.1)	0.432
Female	41 (41.4)	9 (52.9)	
Histology, *n* (%)			
Ad including			0.094
Ad + SCLC	75 (75.8)	8 (47.1)	
Ad + Sq			
Sq	16 (16.1)	7 (41.2)	
NSCLC	8 (8.1)	2 (11.7)	
Stage‐n. (%)			
IIIA,B	20 (20.2)	5 (29.4)	0.394
IVA	32 (32.3)	2 (11.7)	
IVB	47 (47.5)	10 (58.9)	
Tumor location 1, *n* (%)			
Central	34 (34.3)	0 (0)	<0.05
Peripheral	65 (65.7)	17 (100)	
Tumor location 2, *n* (%)			
Right			0.7
Upper and Middle	29 (29.3)	4 (23.5)	
Lower	24 (24.2)	4 (23.5)	
Left			
Upper	24 (24.2)	3 (17.7)	
Lower	22 (22.3)	6 (35.3)	
Maximum diameter of tumor(mm)			
Median (range)	40.3 (14.7–102.3)	49.8 (24.6–85.6)	0.076
Tumor nuclei content (%)			
Median (range)	50 (10–90)	70 (20–80)	<0.05
Necrosis tissue content (%)			
Median (range)	10 (0–90)	5 (0–90)	0.086
Tissue area (mm^2^)			
Median (range)	1.7 (0.2–3.6)	5.6 (0.6–11.5)	<0.001
DNA(μg/μl)			
Median (range)	0.08 (0.01–0.33)	0.08 (0.01–0.17)	0.382
RNA(μg/μl)			
Median (range)	0.02 (0.01–0.28)	0.01 (0.01–0.11)	0.11
DNA/Tissue are (μg/μl/mm^2^ × 10^−2^)			
Median (range)	1.53 (0.12–16.7)	0.68 (0.25–1.10)	<0.001
RNA/Tissue are (μg/μl/mm^2^ × 10^−2^)			
Median (range)	0.42 (0.09–14.8)	0.14 (0.05–2.65)	<0.001
NGS success rate	82/99 (82.8)	12/17 (70.6)	0.312
DNA‐based NGS	88/99 (88.9)	16/17 (94.1)	1.000
RNA‐based NGS	90/99 (90.9)	13/17 (76.5)	0.098

## 4 DISCUSSION

In this study, we compared sampling methods for lung cancer genome analysis through LC‐SCRUM‐Asia, the first nationwide lung cancer genome screening project in Japan. Although our results revealed that surgical biopsy is the best method to obtain samples for successful NGS analysis, fluid samples showed comparably high NGS success rates. For TBB specimens, it was found that tissue confirmation by assessing tumor cells from pre‐ and post‐submitted samples is a meaningful approach to increase NGS success rate when using fresh frozen TBB specimens. Thus, choosing TBB, EBUS‐TBNA, or CT‐guided biopsy for each case depends on the localization of the tumor.

NGS analysis on TBB samples should be performed on at least two or three biopsy specimens with 5.0‐mm diameter forceps when using fresh frozen specimens. Although this study was performed at LC‐SCRUM‐Asia and the protocol calls for submission of fresh frozen specimens, tissue confirmation can increase the likelihood of success even with fresh specimens that are difficult to assess for tumor cell content before sample submission. In addition, indirectly assessing the presence of tumor cells by performing ROSE at each biopsy may be important in assisting tissue confirmation; however, in cases where the tumor is centrally located, even if tumor cells are confirmed by ROSE, the volume of the tumor may not be sufficient and the risk of re‐genome biopsy may increase significantly. In such cases, it is desirable to consider using EBUS‐TBNA instead of TBB.

For peripherally located lesions, CT‐guided biopsy may be easier and more reliable than TBB for sampling. Of the 14 patients who underwent re‐genome biopsy, two underwent CT‐guided biopsy. CT‐guided biopsy can obtain significantly larger samples than TBB, but the success rate of NGS analysis is similar. RNA‐based NGS also tends to be inferior to TBB. In the case of CT‐guided biopsy, it cannot be assumed that submitting samples more than twice used in this study improves the success rate of NGS analysis, considering the size of the samples collected and the amount of nucleic acid yields obtained from the samples. In the case of CT‐guided biopsy, this protocol may require some additional effort, such as immediate nucleic acid extraction after sampling.

Our findings suggest that fluid samples are the best choice for NGS analysis because of their ease of collection and the success rate of NGS. The LC‐SCRUM‐Asia protocol requires submission of 100 ml of fluid samples prior to treatment; if malignant tumor cells are identified, NGS analysis of the pellet from fluid samples will proceed. Although studies have reported NGS analysis of malignant pleural effusion (MPE),^31^, ^32^, ^33^, ^34^ their NGS success rates are not as high as ours. The reasons for this may be that cell blocks made from MPE are used for analysis,^31^ supernatants of MPE are used for analysis,^32^, ^33^, ^34^ and the amount of samples collected is not clearly defined. Our results suggest that the use of fresh fluid samples for NGS analysis is useful for the rapid and simple identification of genome profiles of advanced staged NSCLC. Another study in LC‐SCRUM‐Asia reported NGS success rates as high as 98.7% in the analysis of cytology samples from transbronchial brushing samples during TBB.^19^ The use of fluid samples for NGS analysis seems promising. However, the advantage of surgical biopsy samples is that they can be frozen or stored as paraffin‐embedded samples for future analysis. When a new gene panel is approved in the future, it can be reanalyzed, and when a new biomarker utilizing NGS emerges, the presence of a surgical biopsy specimen will ensure sufficient tissue volume to support such analysis. This is a significant advantage over other sampling methods. A second NGS analysis was reported to identify a targetable driver mutation missed in the first NGS analysis.^35^, ^36^ If surgical biopsy is possible, it should be prioritized. The construction of biobank using surgical biopsy samples is an important material source in future cancer research and medical care.^37^, ^38^

Sampling methods other than TBB are more likely to result in biopsies from metastatic sites. In these cases, the question is whether the profile of the gene mutation in the primary tumor is the same as that in metastatic and primary tumors. In a comprehensive analysis of advanced colorectal cancer and an evaluation of the concordance rate of a few driver mutations between primary and metastatic lesions, a concordance rate of approximately 95% was found when focusing on *KRAS* and *BRAF* hot point mutations.^39^ Application of evolutionary and population–genetic approaches to interpret genomic data has shown that tumor progression is not necessarily sequential but can occur in a punctuated manner.^40^, ^41^ As far as searching for hot point mutations of strong driver mutations associated with the root of cancer development is concerned, it is expected that even the genome profile of metastatic lesions sufficiently reflects targetable driver mutations at the primary site. However, as more information on multiple genetic mutations and clinical application of whole exome sequence data becomes available, discordance rates will increase, and a discussion will be required on whether genomic analysis should be performed from primary or metastatic sites.^42^, ^43^

Our study has a few limitations. First, the exact amount of tumor collected by each sampling method is unclear; an amount is specified only for fluid samples. Undoubtedly, a larger volume of specimens can be obtained with surgical biopsy than with other methods, but sufficient tissue volume for NGS analysis has not been evaluated. In TBB, since the number of times the sample are collected is not strictly determined and the number varies depending on the amount of sample ascertained with the naked eye during examination. In EBUS‐TBNA and CT‐guided biopsy, sampling frequency is determined to be approximately two times. However, in TBB, the optimal sampling frequency cannot be identified in this study, and the optimal TBB sampling method cannot be proposed for NGS analysis because it depends on the results of ROSE during the test. Second, because this study was conducted in a relatively small number of patients at a single institution, no general conclusion can be drawn from the results. The method of sedation used in bronchoscopy, whether to use an echo‐guide, and how to select a sampling method are not specified in the prior protocol. It is not an exact comparison of the sampling method. Third, adverse events associated with each sampling method were not investigated in this study. Although there were no fatal adverse events related to each method, it is essential to select the sampling method by considering complications and adverse events associated with each method, including the time required to introduce treatment after each sampling.

In conclusion, we compared sampling methods for the successful pre‐treatment NGS analysis in LC‐SCRUM‐Asia in patients with advanced lung cancer. First, it is important to discern which sampling method is the best from the image and patient condition to avoid the re‐genome biopsy. If obtaining the sufficient amount of sample by TBB for NGS analysis is difficult, other methods should be considered. Our results suggest that EBUS‐TBNA may be preferable to TBB for centrally located lesions. CT‐guided biopsy is recommended for peripheral samples; however, the problem is that the amount of nucleic acids recovered and the success rate of NGS analysis are not good for the size of the sample. The use of fluid samples is less invasive and has a high success rate. Although surgical biopsy may be applicable to fewer patients, they are most desirable in terms of tissue preservation and success rates, but the high degree of invasion is a concern. NGS analysis is indispensable in the treatment of advanced lung cancer. How quickly and accurately a patient can be analyzed before treatment is the key to success. In patients with advanced lung cancer, it is important to deliver treatment as soon as possible. Moreover, to avoid the risk of re‐genome biopsy, it is important to carefully consider which sampling method is best in advance. The special attitude as a genome biopsy for the success of the NGS analysis is required instead of the biopsy for the diagnosis until now.

## CONFLICT OF INTEREST

Dr. Kunimasa reports grants from the Japan Society for the Promotion of Science (Grant no. JP19K176974), Takeda Science Foundation, The Osaka Medical Research Foundation for Intractable Diseases, and honoraria for lecture from AstraZeneca, Chugai Pharma and Novartis. Dr. Matsumoto reports grants from MSD, Merck, Chugai Pharma, Novartis, Lilly, honoraria for lecture from Novartis pharma, Dr. Nishino reports a grant from Nippon Boehringer Ingelheim and honoraria for lecture from Chugai Pharma, AstraZeneca, Nippon Boehringer Ingelheim, Eli Lilly Japan, Roche Diagnostics, Novartis, Pfizer Merk; Dr. Tamiya reports grants from Ono Pharmaceutical, Bristol‐Myers Squibb, Boehringer Ingelheim and honoraria for lecture from Taiho Pharmaceutical, Eli Lilly, Asahi Kasei Pharmaceutical, MSD, Boehringer Ingelheim, AstraZeneca, Chugai Pharmaceutical, Ono Pharmaceutical, Bristol‐Myers Squibb; Dr. Goto reports grants from AstraZeneca, Bristol‐Myers Squibb, Chugai, and Ono, during the conduct of the study; grants from AbbVie, Astellas, Boehringer Ingelheim, Daiichisankyo, Eisai, Kyowa Hakko Kirin, Ignyta, Janssen, Life Technologies, Lilly, Loxo Oncology, Merck Serono, MSD, Novartis, Oxonc, Pfizer, SRL, Sumitomo Dainippon, Sysmex Corporation, RIKEN GENESIS, Roche, Taiho, Takeda, honoraria for lecture from AbbVie, AstraZeneca, Boehringer Ingelheim, Bristol‐Myers Squibb, Chugai, Daiichisankyo, Life Technologies, Lilly, Ono, Merck Serono, MSD, Nippon Kayaku, Novartis, Pfizer, RIKEN GENESIS, Roche, SRL, Taiho, Takeda, outside the submitted work. Dr. Kumagai reports grants from Ono Pharmaceutical, MSD K.K., Chugai Pharceutical Co. Ltd、AstraZeneca K.K. Takeda Pharmaceutical Companey Limited. Regeneron Pharmaceuticals, Inc. Merck Serono Co., Ltd. Pfizer Japan Inc. Taiho Pharmaceutical Co.,Ltd. Nippon Boehringer lngelheim Co., Ltd. Eli Lilly Japan K.K. Novartis Pharma K.K. AbbVie GK., Delta‐Fly Pharma, Inc. The Osaka Foundation for The Prevention of Cancer and Life style‐related Diseases (Public Interest Incorporated Foundation), and personal fees from Ono Pharmaceutical, AstraZeneca K. K., Taiho Pharmaceutical Co. Ltd., MSD K.K., TEIJIN PHARMA LIMITED, Novartis Pharma K.K. Nippon Boehringer Ingelheim Co., Ltd. Eli Lilly Japan K.K. Pfizer Inc., Chugai Pharceutical Co. Ltd., Bristol‐Myers Squibb K.K.

## AUTHOR CONTRIBUTIONS

Kei Kunimasa: Conceptualization, Methodology, Investigation, Writing ‐ original draft, Shingo Matsumoto: Conceptualization, Investigation, Methodology, Supervision, Kazumi Nishino: Data collection, Care for patients, Keiichiro Honma: Data collection, Served as pathological specialist, Noboru Maeda, Hanako Kuhara, Motohiro Tamiya, Takako Inoue, Takahisa Kawamura, Toru Kimura, and Tomohiro Maniwa: Data cellection, Jiro Okami: Supervision, Koichi Goto: Conceptualization, Investigation, and Supervision, Toru Kumagai: Supervision.

## ETHICS STATEMENT

The Lung Cancer Genomic Screening Project for Individualized Medicine in Asia is in the UMIN Clinical Trial Registry (UMIN 000036871). This study was approved by the Institutional Review Board at Osaka International Cancer Institute (#19018–3). Informed consents were obtained from the all cases who were enrolled in LC‐SCRUM Asia at our institute.

## Supporting information


**Data S1.** Supporting Information

## Data Availability

All data in the present study are available via the corresponding author (K.K, kei.kunimasa@oici.jp).
