# Supplementary material for: Comparison of sampling methods for next generation sequencing for patients with lung cancer
Source: Cancer Med. 2022 Mar 10;11(14):2744–54. doi: 10.1002/cam4.4632 (PMC9302352; doi:10.1002/cam4.4632)
Supplement: Supplementary file 1 — Data S1. Supporting Information [file CAM4-11-2744-s001.zip › cam44632-sup-0005-TableS3.docx]

**Supplementary Table.3 Analysis of clinical factors for the success of NGS analysis in TBB samples**
