# Supplementary material for: Comparison of sampling methods for next generation sequencing for patients with lung cancer
Source: Cancer Med. 2022 Mar 10;11(14):2744–54. doi: 10.1002/cam4.4632 (PMC9302352; doi:10.1002/cam4.4632)
Supplement: Supplementary file 1 — Data S1. Supporting Information [file CAM4-11-2744-s001.zip › CAM4_4632_Supplementary Figure legends.docx]

**Supplementary Figure legend**

**Supplementary figure.1**

Comparison of DNA and RNA yields (μg/μl) with the failure and success groups in all sampling methods. **P* < 0.05; ***P* < 0.05.

**Supplementary figure.2**

Chest CT images and endobronchial ultrasonography (EBUS) images of representative case series of centrally located lung cancers. EBUS probes images suggested that the probes were inserted into the tumors, but in these cases re-genome biopsy was necessary because TBB failed to obtain sufficient tissue for NGS analysis.
